# RSM process optimization of biodiesel production from rapeseed oil and waste corn oil in the presence of green and novel catalyst

**DOI:** 10.1038/s41598-022-20538-4

**Published:** 2022-11-16

**Authors:** Taiebeh Tamoradi, Ali Reza Kiasat, Hojat Veisi, Valiollah Nobakht, Bikash Karmakar

**Affiliations:** 1 Department of Chemistry, Faculty of Sciences, Shahid Chamran University of Ahvaz, Ahvaz, Iran; 2 Department of Chemistry, Production Technology Research Institute-ACECR, Ahvaz, Iran; 3 Department of Chemistry, Payame Noor University, Tehran, 19395-4697 Iran; 4 Department of Chemistry, Gobardanga Hindu College, 24-Parganas (North), Gobardanga, India

**Keywords:** Environmental sciences, Chemistry, Nanoscience and technology

## Abstract

In the scenario of global warming and pollution, the green synthesis and use of biodiesel has acquired utmost priority. Due to several limitations of homogeneous catalysis, organobase immobilized heterogeneous catalyzed production of biodiesel has come out as a favored route. The present report demonstrates the design and synthesis of *Peganum harmala* spice seed extract modified GO-CuFe_2_O_4_ (SSE@GO-CuFe_2_O_4_) nanocomposite as an organobase functionalized high surface area magnetic nanocatalyst. *Pistachio* leaves were used in the green reduction of precursor salts to synthesize CuFe_2_O_4_ NPs. The as-synthesized nanomaterial was characterized physicochemically by Fourier-transform infrared spectroscopy (FT-IR), scanning electron microscopy (SEM), energy dispersive X-Ray analysis (EDX), elemental mapping, transmission electron microscopy (TEM), X-Ray diffraction (XRD), thermogravimetric analysis (TGA) and vibrating sample magnetometer techniques (VSM). Subsequently, the catalyst was explored in the efficient synthesis of biodiesels by trans-esterification of two substrates, the rapeseed oil and waste corn oil. The optimum conditions for biodiesel production were determined through response surface methodology based on Box–Behnken design including the study of calibration curves and 3D contour plots. Easy separation and workup, use of green medium, excellent reused for several times and short reaction time are outstanding benefits of this study.

## Introduction

In recent times, the extravagant consumption of natural energy resources, the ever-increasing environmental pollution and the resulting global warming is a serious concern worldwide. Industrial development and upgradation in the standard of living in the society has made the circumstances more dreadful^1–3^. The storage of fossil fuel has been literally going to be exhausted and this has persuaded the scientists to rethink about the generation of non-conventional energy from renewable sources and also developing advanced technological methodologies that would retard the energy consumption and reduces hazardous wastes thereby leading to sustainability^4–8^. The extensive research has demonstrated that biofuels, more precisely, the biodiesels could be the best possible solution as a promising and featured green fuel towards alternant energy^9–12^. In contradiction to fossil fuels, biodiesels are beneficial with regards to its higher oxygen content, devoid of sulphur and carcinogen content, reproducibility, inexpensive and abundant precursors, biodegradability, eco-friendliness, minimally toxic and very low exhaust emissions to the environment, although they are having equivalent calorific value to fossil fuels while burning^13–17^. In addition, biodiesels possess higher combustion efficiency and cetane number and also having outstanding lubricity. Due to higher flash point, the biodiesel is also much safe to store, handling and transport than conventional fuels^18–20^. Although, the bio-diesels are costly now-a-days than petro fuels, in view of these favorable issues, a number of research groups around the world are engaged in developing the synthesis protocol in order to cut down the production cost.

Usually, biodiesels are produced following different pathways like micro emulsion, pyrolysis and transesterification and among them the last one is most facile, handy and sustainable^9,10^. Transesterfication involves the alcoholysis, mainly methanolysis of esters, being obtained from different feedstock like waste cooking oil, bio-wastes, agricultural wastes, animal fats, vegetable oil and also from the natural resources like non-edible jatropha oil, canola oil, neem oil, tea oil, cotton oil, tobacco oiletc and edible soybean oil, palm oil, coconut oil, castor oil^21–34^.

In the recent years, quite a number of researcher have accounted the proficient synthesis of methyl ester biodiesel applying different homogeneous or heterogeneous acid or base catalysts. Keeping in mind of recent trend for green and sustainable protocols, heterogeneous catalysts has proven their dominance over the former in all domains of catalysis^35–51^. In this context, different architectured nanomaterials have come upon as the protagonist of heterogeneous catalysts. Particularly, the biomolecularly modified magnetic nanoparticles (NP) have garnered significant attention these days based on their high surface to volume ratio, larger number of surface atoms or active sites, exceptional mechanochemical and thermal stability, biocompatibility, effortless isolation from the system by just using an external magnet and reusability with consistent reactivity^52–65^.

**Figure 1:**
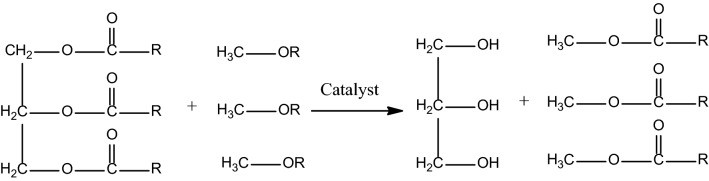
General reaction scheme for transesterification.

With all these inputs, this work demonstrate the novel engineered synthesis of *Peganum harmala* spice seed extract (SSE) immobilized over graphene oxide (GO) and CuFe_2_O_4_ nanocomposite and subsequent application of the material in the transesterification waste corn oil and rapeseed oil towards the synthesis of bio-diesels^65^. The *Peganum harmala,* commonly known as Syrian or African rue, linked to Zygophyllacea family, is a wild flowering plant and grows abundantly in Middle East and North African countries. The seed extract contains large extent of β-carbolines such asharmaline, harmine, harmalol, harmol, tetrahydroharmine, and the quinazoline derivatives like vasicinone and deoxyvasicinone^66–68^. Both these nitrogen containing heterocyclic scaffolds are basic in nature which has been the driving force behind its excellent catalytic potential in trans-esterification. CuFe_2_O_4_ doped GO was used as the base matrix in view of exploiting the high surface area of GO as well as the easy magnetic reusability due to ferrite. Green pistachio leaf extract was used as the green reductant in the production of GO-CuFe_2_O_4_ composite^65^. To the best perception, this report on the biodiesel synthesis (Fig. 1) from waste corn oil and rapeseed oil catalyzed over green synthesized magnetic nanocomposite (SSE@GO-CuFe_2_O_4_) is unprecedented.

Optimization of the reaction parameters such as molar ratio of methanol to oil, amount of catalyst, volume of water usedand reaction time through Response Surface Methodology (RSM)is another important wing of this study. It is a combined statistical and mathematical approach having wide applicability^69–71^. The optimization protocol involves the drawing of contours and curves in 2-D or 3-D planes and from the corresponding shapes the interaction between the variables are illustrated. The effects of the parameters and their interactions were studied through Analysis of variance (ANOVA).

## Experimental

This section includes several segments including material, methods and the experimental details.

### Materials and methods

All the chemicals and solvents were procured from Sigma-Aldrich and Merck from USA and Germany and used without further purification (99.9% pure). NaNO_3_, KMnO_4_, CuCl_2_·2H_2_O, FeCl_3_·6H_2_O, NaOH, MeOH and EtOH were used for this project. Good quality rapeseed oil and waste corn oil were collected from market. The particle size and morphology were determined by measuring SEM using FESEM-TESCAN MIRA3 instrument (Czeck Republic). FT-IR analysis was performed on KBr pellets in a BRUKER spectrophotometer (model VRTEX 70, Germany). Powder X-ray diffraction (XRD) was investigated using Cu Kα radiation (λ= 1.54060 Å). The outputs were processed through origin software. Magnetic properties of the catalyst were studied on a Vibrating Sample Magnetometer (VSM) MDKFD, USA. Gas chromatographic analysis was carried out using a GC-2014 chromatograph from Shimadzu, Japan equipped with flame ionization detector (FID) and capillary column (Omegawax,30 m/0.25 mm/0.25 mL). The detector and injector were set at 250 °C and 260 °C, respectively. The oven program was set as 200 °C for 5 min, and then the temperature was increased to 260 °C at a ramping rate of 20 °C/min and kept constant 260 °C for 6 min. Helium was used as a carrier gas at 2mL/min flow-rate. *Peganum harmala* spice seed and *Pistachio* leaves were collected from Khuzestan province mountains and identified and approved by Dr. Tamoradi based on morphological and anatomical characters listed in textbook of plant taxonomy.

### Synthesis of GO

Graphene oxide (GO) was synthesized by oxidizing graphite powder following a modified Hummers method^65^. In the typical procedure 2.0 g graphite powder, 1.0 g NaNO_3_, 6.0 g KMnO_4_ were mixed to 60 mL concentrated H_2_SO_4_ (98%) and vigorously stirred covering in an ice bath for 2 h. It resulted a blackish-green paste which was cooled 35 °C water bath and maintained at this temperature for 2 h. 150 mLdistilled water was then slowly added to it which raised the reaction temperature to 100 °C. The mixture was cooled to 60 °C, 10 mL H_2_O_2_ (30%) was then added to the mixture and further stirred for another 2 h. The thick solid obtained was filtered and thoroughly washed with hydrochloric acid (5%) and distilled water for several times until neutral pH. It was dried under vacuum at 60 °C. The GO was exfoliated by sonication for 2 h in water.

### Preparation of Pistachio leaf extract

Fresh Green pistachio leaves were washed thoroughly with distilled water. 10 g of the leaves were boiled in 100 mL of DI water for 15 min. The mixture was then cooled and filtered through Whatman filter paper No. 1. The filtrate so obtained was stored in refrigerator at 4 °C for further use.

### Green synthesis of GO-CuFe_2_O_4_ nanocomposite

1.0 g GO was dispersed in 100 mL DI water by sonication. A mixture of two salts CuCl_2_·2H_2_O (11.43 mmol) and FeCl_3_·6H_2_O (22.8 mmol) (molar ratio of Cu^2+^/Fe^3+^= 1/2) in 10 mL DI water was added to the dispersion. The pistachio leaf extract (5 mL) was then slowly added to it followed by a NaOH solution (0.1 M) under stirring till it became strongly alkaline (pH=11). After stirring for 2 h, a thick white solid precipitate was obtained which was separated using a magnet. The material was washed with deionized water followed by EtOH and dried in a vacuum oven at 70 °C overnight.

### Synthesis of SSE@GO-CuFe_2_O_4_ nanocomposite

**Figure 2:**
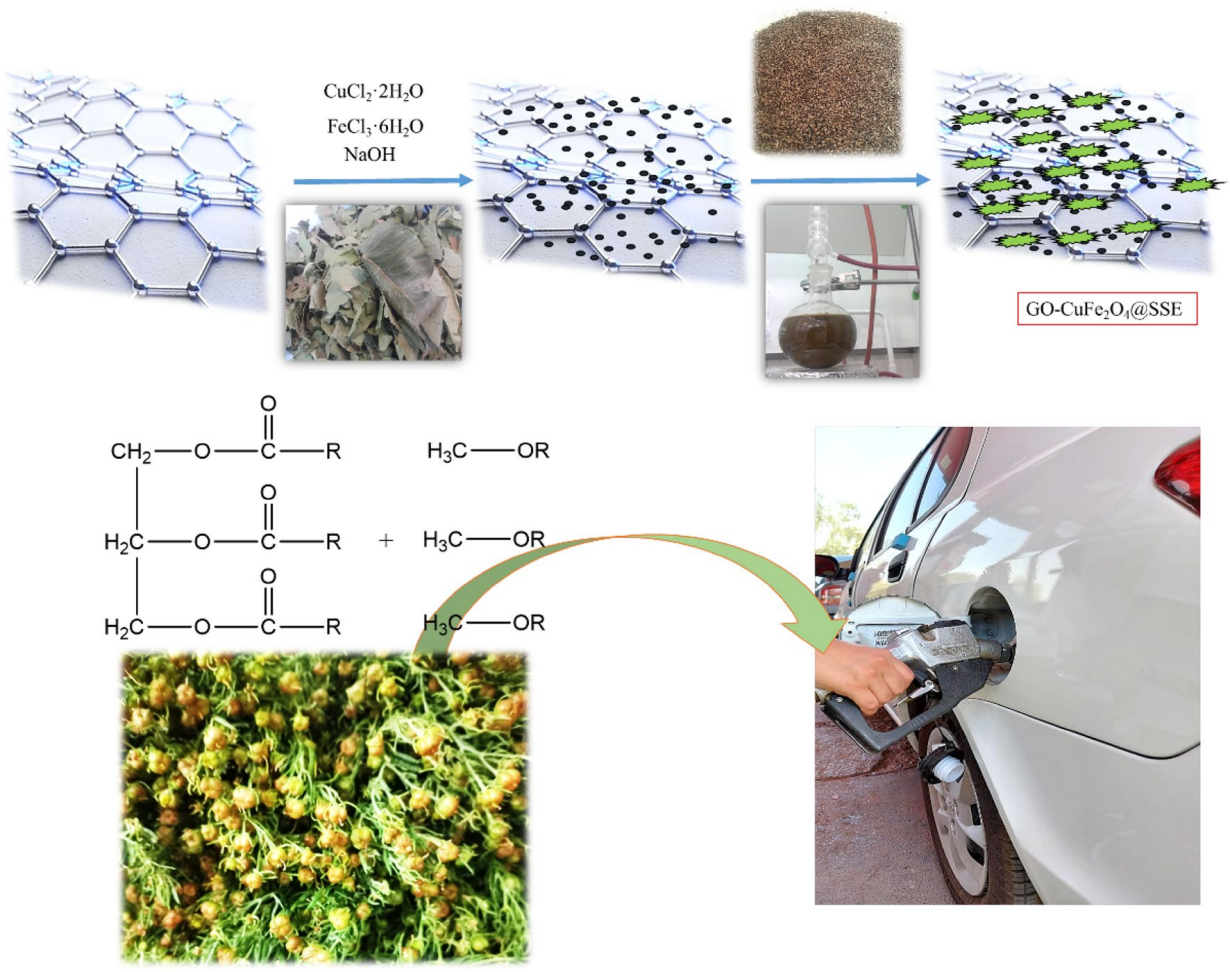
Schematic preparation of SSE@GO-CuFe_2_O_4_ nanocomposite.

Fresh spice seeds were collected and washed thoroughly with double-distilled water before use. 30 g of the seeds was added to 100 mL of deionized water/ethanol (1:1) and boiled for 15 min in a water bath. It was then cooled and filtered through Whatmann-1 filter paper. A clear filtrate was obtained as the SSE extract. In a separate flask 0.5 g of GO-CuFe_2_O_4_was dispersed in 50 mL by sonication for 20 min. The spice seed extract was then added to it and stirred for 24 h at room temperature. The precipitate so obtained was separated by magnetic decantation and washed several times with deionized water. It was dried in a vacuum oven at 40 °C for 12 h to afford the SSE@GO-CuFe_2_O_4_ nanocomposite (Fig. 2).

### Transesterification of fatty acids

50 g oil was used in the esterification reaction for 13 experimental runs required in RSM study. For each set, oil was transferred into the reaction flask and preheated in an oil bath up to the reaction temperature. MeOH and the catalyst was then added to it and the mixture was stirred vigorously. After completion (by GC), the catalyst was isolated magnetically and the mixture was allowed to settle in a separating flask for 6 h to separate the biodiesel phases from the byproduct glycerol and methanol-water mixture. The isolated biodiesel layer was further washed with distilled water at 40 °C to remove the residual impurities and concentrated on rotary evaporator at 70 °C. The glycerol is also separately purified for use in its traditional applications (pharmaceutical, cosmetics and food industries).

## Results and discussion

### Characterization of prepared catalyst

The catalyst has been prepared by co-precipitation method over the high surface area of GO. Pistachio leaves extract was used for green reduction of the precursor salts into the regularly grown magnetic NPs and also to stabilize them. In order to introduce a basic flavor over the modified magnetic core, the *Peganum harmala* seed extract was immobilized thereon. The as-synthesized green nanocomposite was characterized by a wide range of physicochemical techniques like FT-IR, SEM, EDX, TEM, elemental mapping, VSM, XRD and TGA.

**Figure 3:**
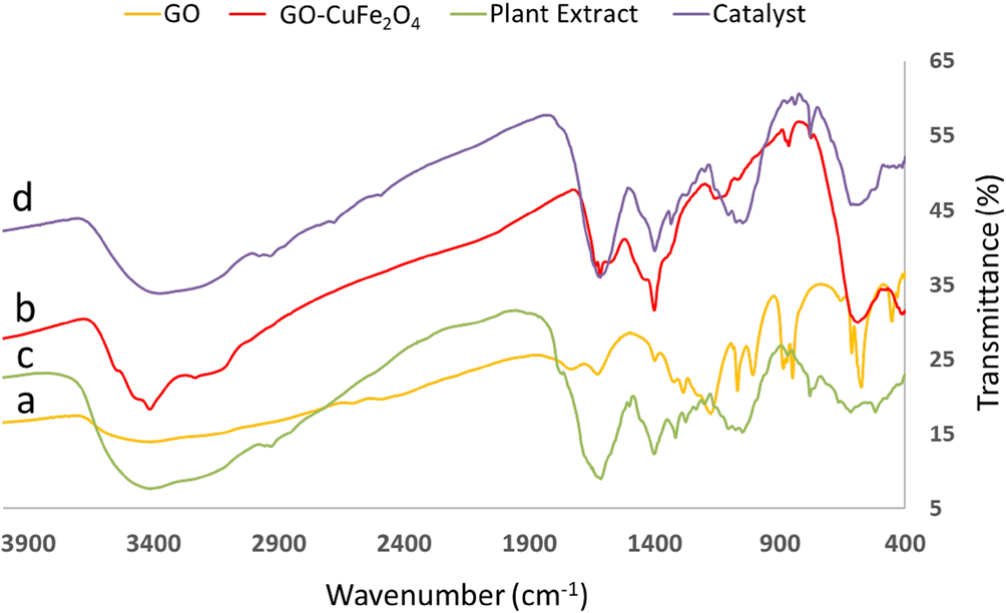
FT-IR spectra of GO (**a**), GO-CuFe_2_O_4_ (**b**), plant extract (**c**) and SSE@GO-CuFe_2_O_4_ (**d**).

Fig. 3 depicts the FT-IR spectra of GO, GO-CuFe_2_O_4_, Peganum plant extract and the final SSE@GO-CuFe_2_O_4_ in order to explain the stepwise construction. In Fig. 3a the FT-IR spectrum of GO is shown where the characteristic peaks appeared at 1052 cm^−1^, 1402 cm^−1^, 1735 cm^−1^ and a broad range peak at 3100-3500 cm^−1^ corresponding to C-O stretching, the C-OH stretching, carboxyl stretching, C=O stretching and the combined O-H broad and the intercalated water stretching vibrations^72,73^. The spectrum of GO-CuFe_2_O_4_ composite (Fig. 3b) could be detected by the precise peaks for Cu-O and Fe-O stretching vibration in CuFe_2_O_4_spinel, being observed at 408 and 584 cm^−1^ respectively, in addition to all the peaks of GO. This justifies the successful mixing of CuFe_2_O_4_ NPs with GO. Fig 3c represents the *peganum harmala* plant extract showing a broad range of vibrations in the range of 3000-3500 cm^−1^ attributed to overlapped phenolic O-H and N-H bonds. There are also characteristic vibrations due to carbonyl and carboxyl functions. Finally, the spectrum of SSE doped GO-CuFe_2_O_4_ nanocomposite is shown in Fig. 3d, which completely seems to be an amalgam of its component intermediates as well as the plant extract spectrum. This validates an effective overlap or modification of the intermediates over the core NPs.

**Figure 4:**
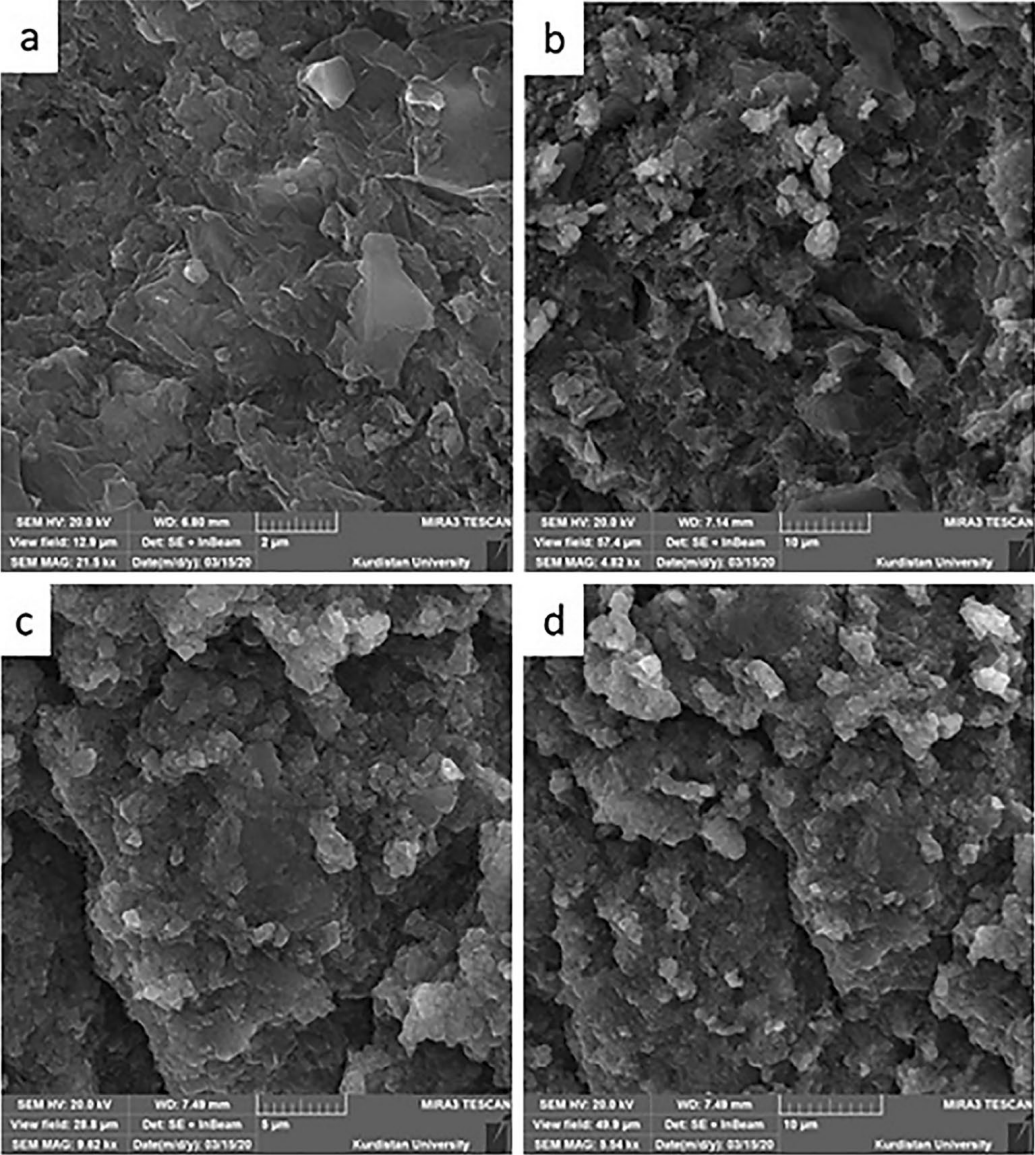
SEM micrograph of GO (**a**), GO-CuFe_2_O_4_ (**b**) and SSE@GO-CuFe_2_O_4_ (**c**,**d**).

In order to assess the comprehensive morphology, shape and texture of the GO, GO-CuFe_2_O_4_ composite and the final SSE@GO-CuFe_2_O_4_nanocomposite, SEM analysis was carried out and the corresponding outcomes are shown in Fig. 4. GO has a typical flaky appearance with a peel like structure (Fig. 4a). Due to large and two dimensional thin surface the GO sheet seems to be wrinkled^53,74^. The SEM micrograph of GO-CuFe_2_O_4_ displays an assembled particle and sheet morphology, as shown in Fig. 4b. The final material appears almost analogous to Fig. 4b. The presence of a coating of the plant extract can be anticipated over the composite molecule (Fig. 4c,d).

**Figure 5:**
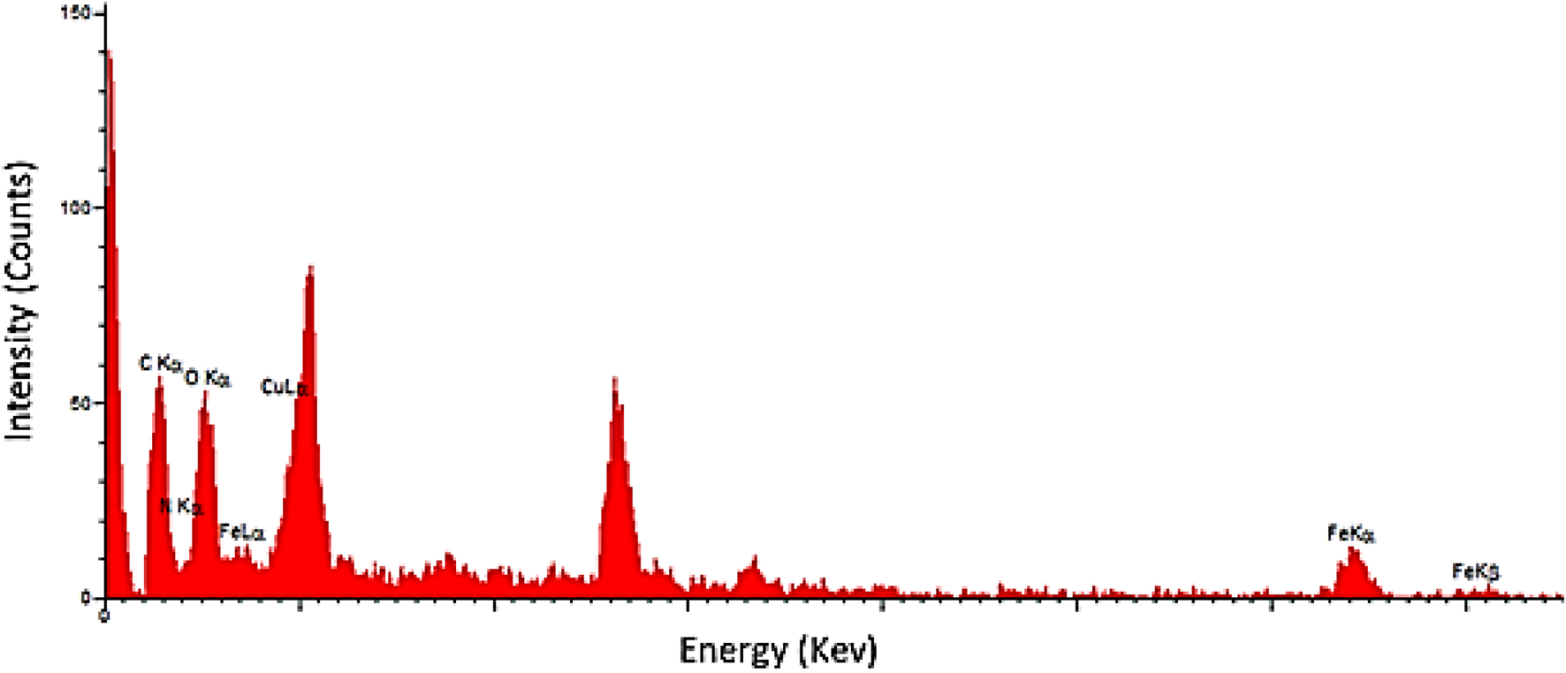
EDX analysis of SSE@GO-CuFe_2_O_4_ nanocomposite.

EDX analysis was carried out to determine the molecular composition of SSE@GO-CuFe_2_O_4_nanocomposite, being displayed in Fig. 5. Evidently, it contains Fe and Cu as metallic and C, N, O as non-metallic components. Cu and Fe obviously are corroborated to the CuFe_2_O_4_ NPs. The non-metallic components reveals the association of GO and spice seed extract containing the N-heterocycles^74^. The EDX data were further justified by elemental mapping analysis.

**Figure 6:**
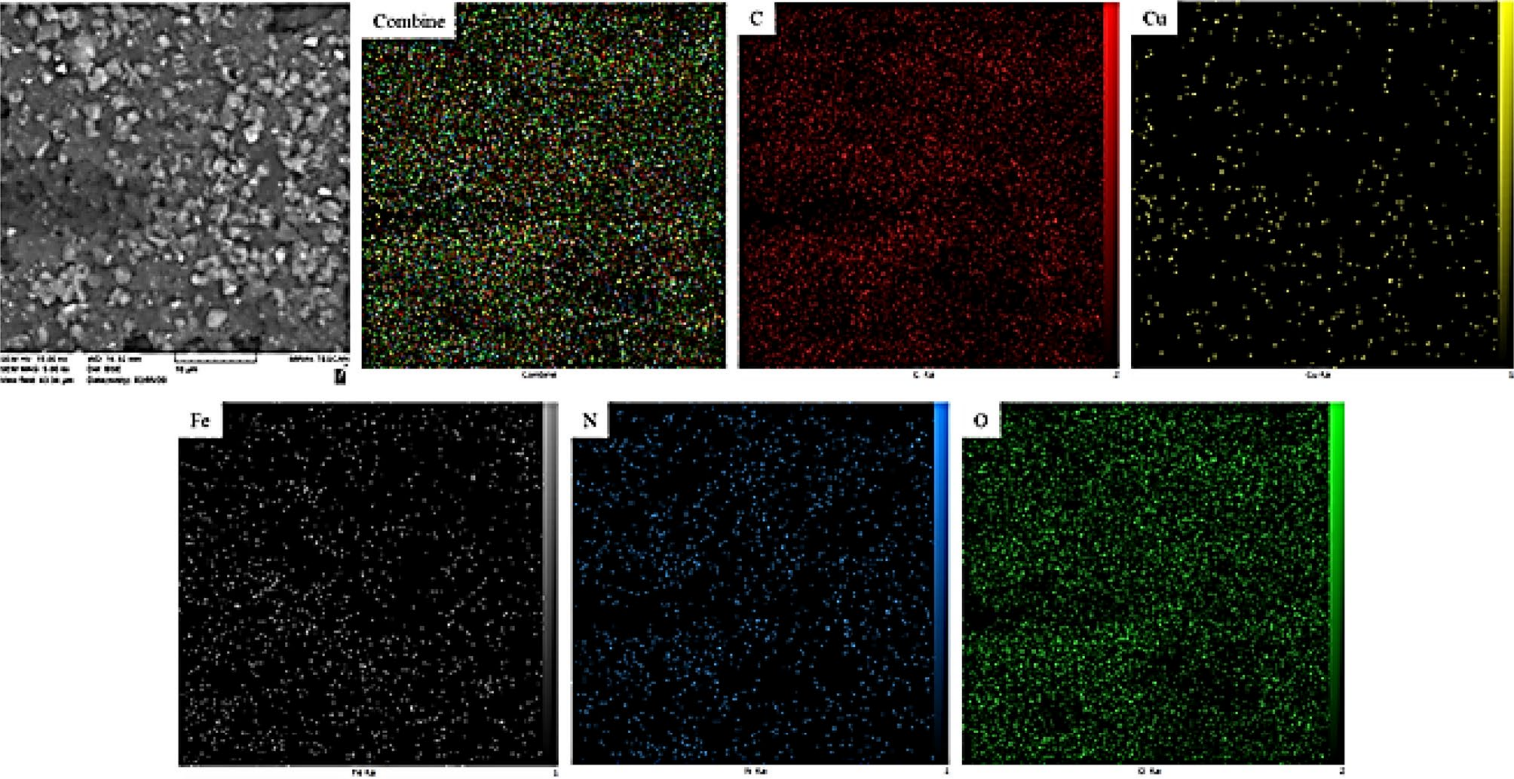
Elemental mapping of SSE@GO-CuFe_2_O_4_ nanocomposite.

X-ray scanning of a section of the SEM image shows the constituting elements which are uniformly dispersed over the high surface matrix (Fig. 6). Homogeneous dispersion of the active species is an utmost important factor in heterogeneous catalysis better catalytic activity^52^.

**Figure 7:**
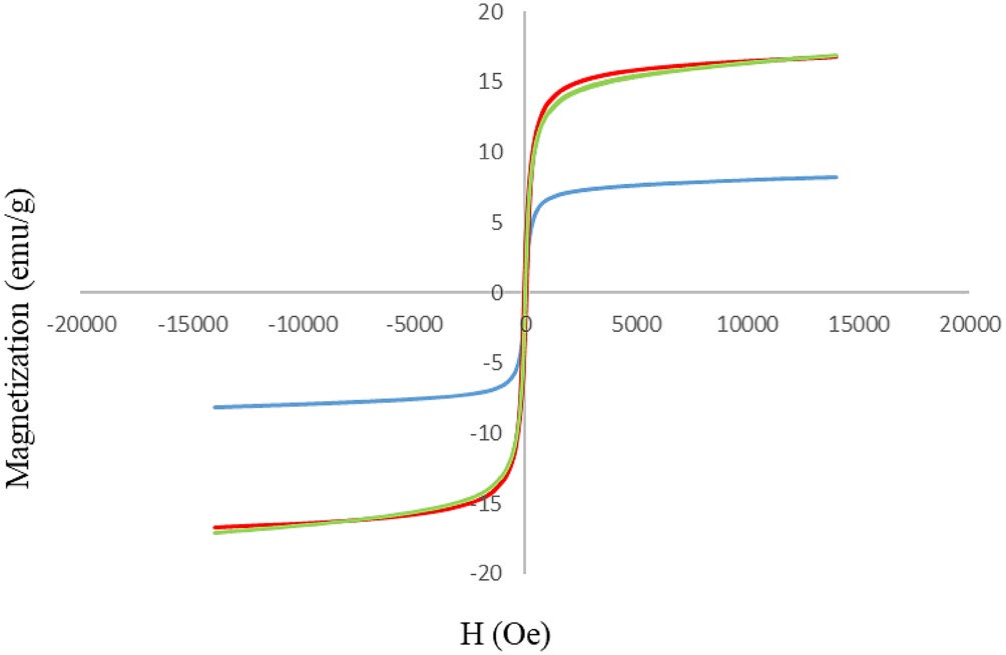
Magnetic hysteresis curves of CuFe_2_O_4_ (red) GO-CuFe_2_O_4_ (green) and SSE@GO-CuFe_2_O_4_ (blue) nanocomposites.

For magnetic core materials, study of magnetism is a necessary measure. Accordingly, this project went through vibrating sample magnetometric (VSM) study for the GO-CuFe_2_O_4_ and SSE@GO-CuFe_2_O_4_ nanocomposites and was compared to the unmodified CuFe_2_O_4_ NPs. The outcome reveals magnetic hysteresis curves as displayed in Fig. 7. Here, the magnetic curve of pure CuFe_2_O_4_ almost overlaps with GO-CuFe_2_O_4_ nanocomposite indicating the insertion of GO into the magnetic NP did not have any magnetic impact. Nature of the curves clearly specify that all the materials are of superparamagnetic behavior. Saturation magnetization (M_s_) values of them were observed to be 15.1, 15.2 and 6.4 emu/g respectively. The diminished magnetism in the final material is predictable due to the incorporation of non-magnetic spice seed extract into the composite of GO-CuFe_2_O_4_^74^.

**Figure 8:**
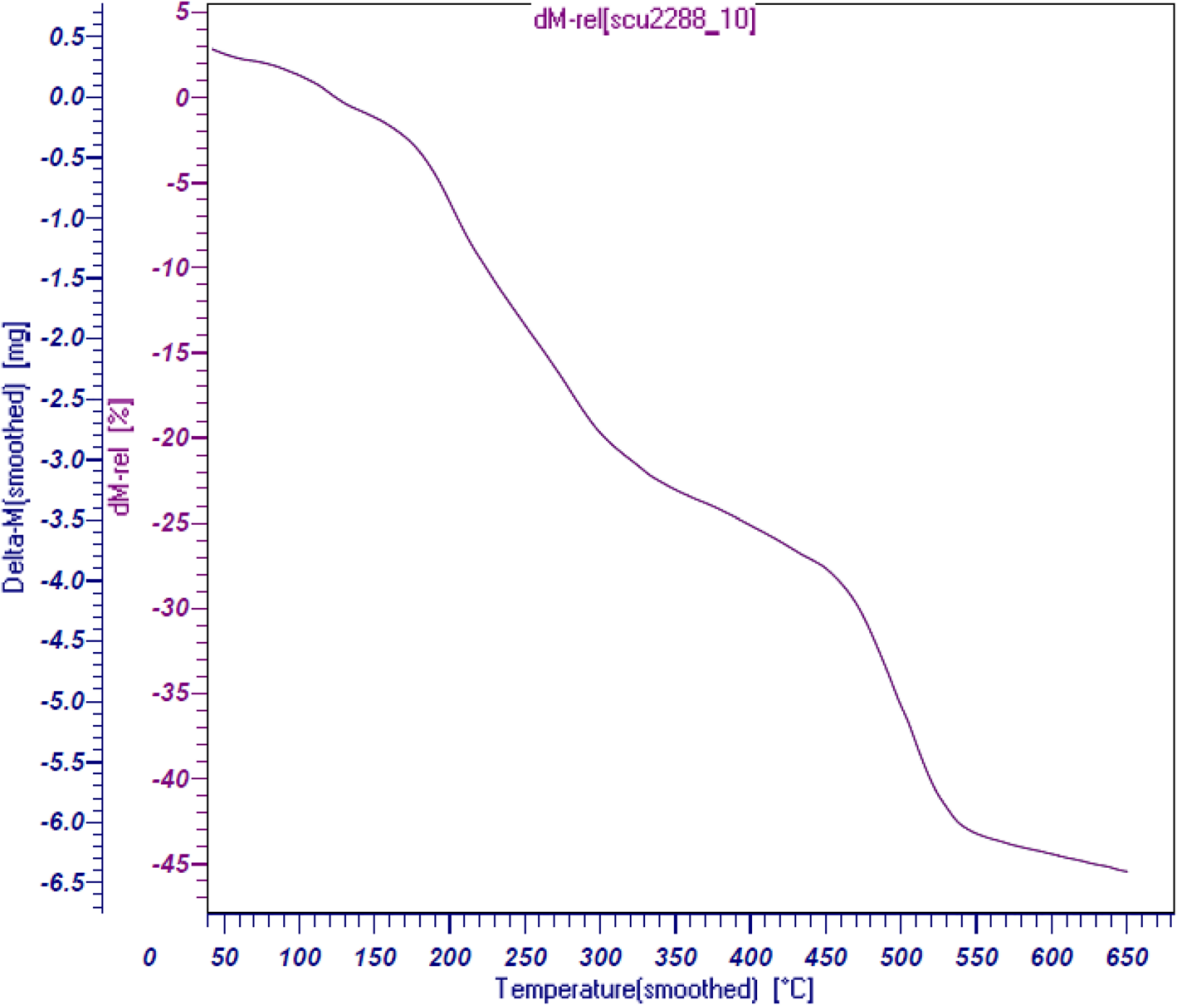
Thermo-gravimetric analysis of the SSE@GO-CuFe_2_O_4_ nanocomposite.

Thermal stability and toughness of the final nanocomposite was estimated by Thermo-Gravimetric Analysis (TGA). The analysis also helps to determine the biomolecular and organic attachments quantitatively. The related curve forSSE@GO-CuFe_2_O_4_ has been presented in Fig. 8. Markedly, it decays continuously starting from 50 °C up to 650 °C with an overall 45% mass loss. An initial 5–6% weight loss occurrstill 150 °C, due to theremoval of moiture and surface hydroxyl groups. Two sharp breaks are detected in the temperature range from 150 °C to 300 °C and 450 °C to 550 °C with a mass loss of 15% each, which are anticipated due to the decomposition of biomolecules of pistachio leaves extract and spice seed extract respectively. This indicates that the catalyst is sufficiently stable for the reaction to carry out^74^.

**Figure 9:**
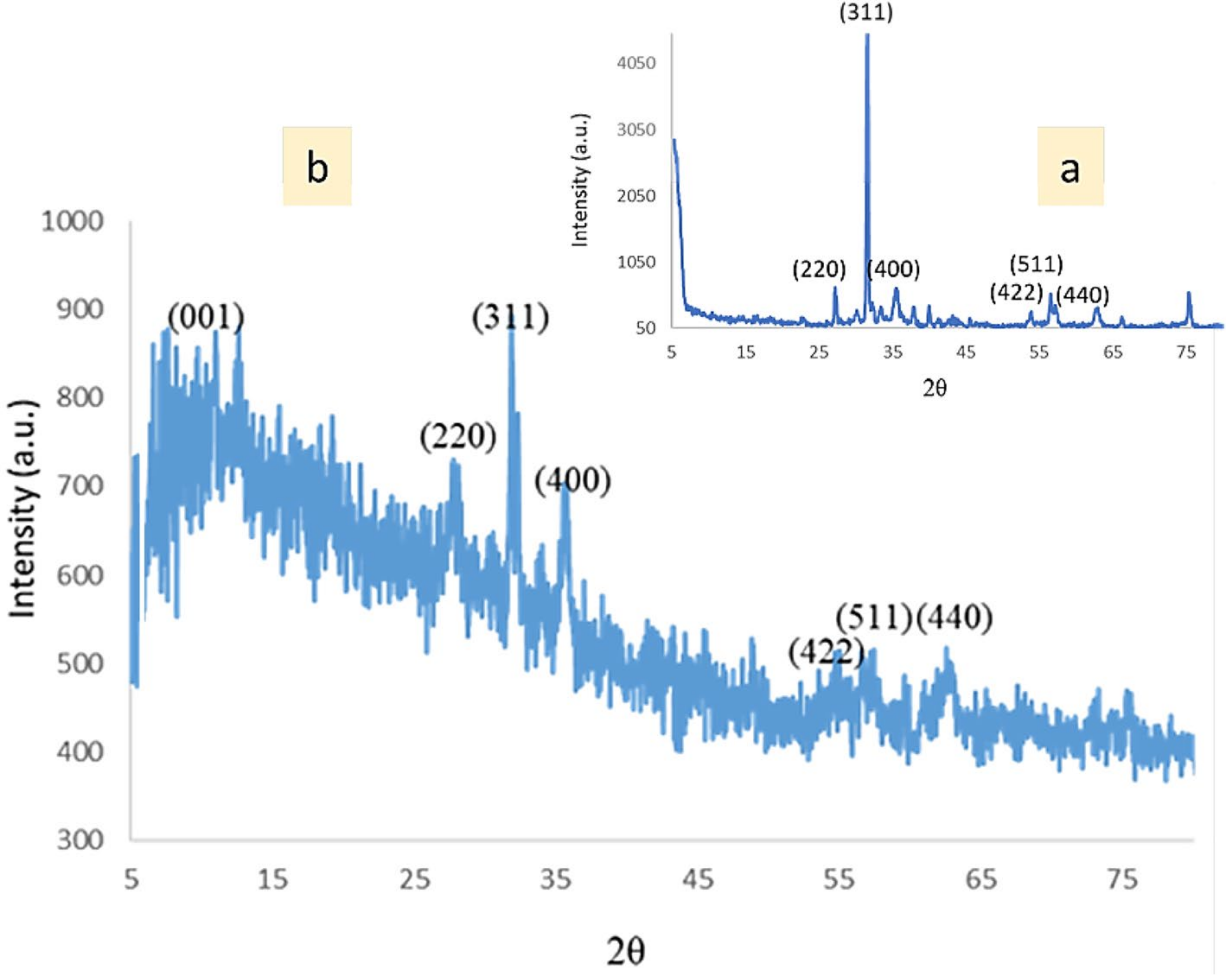
XRD patterns of the CuFe_2_O_4_ (**a**) and SSE@GO-CuFe_2_O_4_ (**b**) nanocomposite.

The crystalline nature and phase structure SSE@GO-CuFe_2_O_4_ nanocomposite was analyzed by XRD study, being presented in Fig. 9. The profile was compared to the diffraction pattern of unmodified CuFe_2_O_4_ NPs (Fig. 9a). The single phase XRD profile clearly reveals that SSE@GO-CuFe_2_O_4_ nanocomposite is a distinct entity and firmly bonded together with all its constituents (Fig. 9b). The broad and weakly crystalline domain in the diffraction angle of 2θ = 10-20º corresponds to the (001) plane of amorphous GO. CuFe_2_O_4_ spinel NPs are detected by the diffraction peaks appeared at 2θ = 28°, 34°, 38°, 55°, 57° and 64° attributed to (2 2 0), (3 1 1), (4 0 0), (4 2 2), (5 1 1), (4 4 0) diffraction planes and matches absolutely as Fig. 9a. Obviously, there occurs no significant change in the crystal structure of core CoFe_2_O_4_ NP phase even after composite formation with GO and further surface modifications^74^.

**Figure 10:**
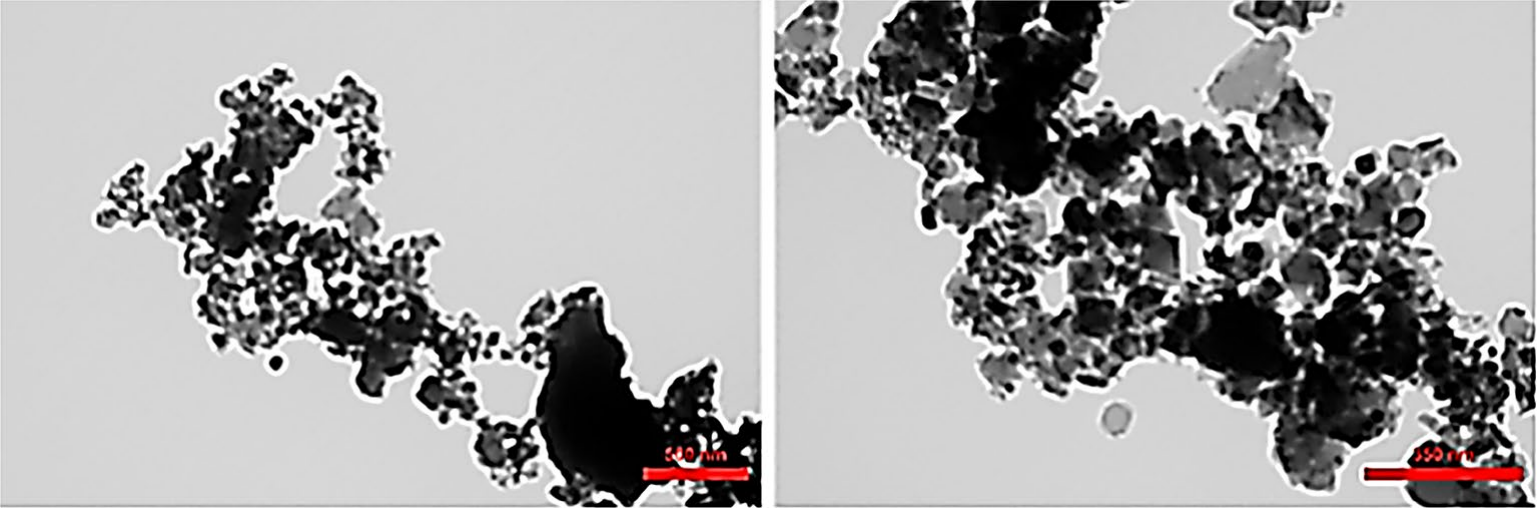
TEM analysis of SSE@GO-CuFe_2_O_4_ nanocomposite.

TEM analysis has been carried out in order to study the particle size and morphology in details. Morphology of SSE@GO-CuFe_2_O_4_ nanocomposite in Fig. 10 confirmed that the hard particles are immobilized on the surface of GO layers. The particle sizes were in the range of 30–40 nm.

### Analysis of reaction data

**Figure 11:**
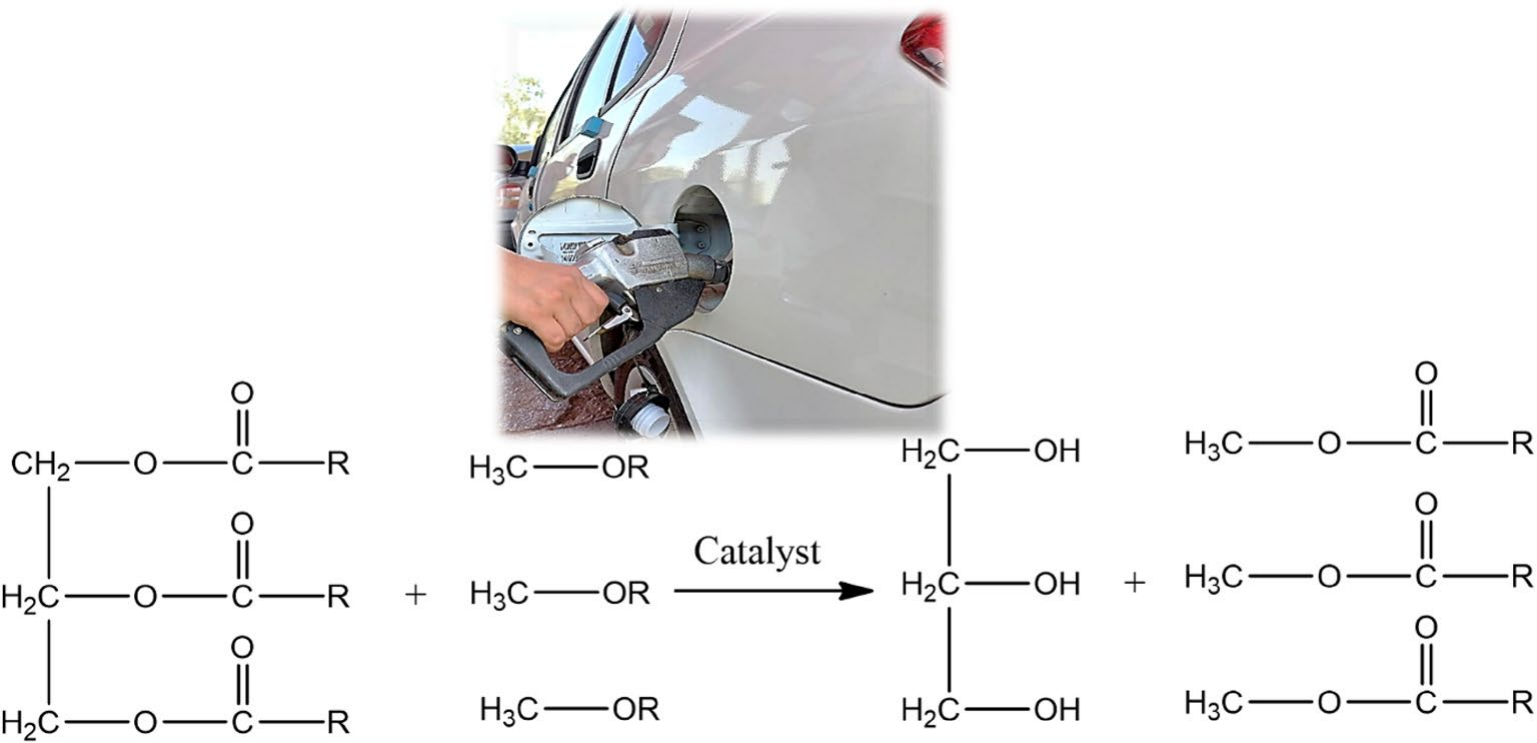
Transesterification of glycerol esters with MeOH over nanomagnetic SSE@GO-CuFe_2_O_4_ catalyst.

Following up with the meticulous physicochemical characterization of SSE@GO-CuFe_2_O_4_ nanocomposite, the next endeavor was to explore its catalytic activity over the synthesis of biodiesels through transesterification of rapeseed oil and waste corn oil (Fig. 11). Here, the reaction protocol has been standardized through response surface methodology (RSM), an advanced mathematical model, other than the typical method of condition optimizations. RSM involves a sequential process that goes through a practical and logical operating reaction process and includes models building, design of experiment and statistics which ultimately stimulates the optimization parameters. RSM is based on Box–Behnken statistical model that leads to the maximum yield of product. In this process, three different reaction parameters, viz., catalyst concentration, methanol to oil molar ratio and time (h), are investigated within a certain range, as documented in Table 1. Subsequently, a series of experiments, usually 13 runs, are conducted to acquire the best optimized result. A mathematical model is applied to validate the experimental results^47^. In this study the catalyst concentration and the methanol to oil ratio were limited within 3 to 8 w/V % and 10 to 16 respectively. Similarly, the reaction time was limited to 4 to 7 h.

**Table 1:** Codes and ranges of three independent variables in RSM design.

	Name	Units	Low	High
A	Catalyst concentration	% w/v	3	8
B	M:O (methanol/oil)	–	10	16
C	Time	h	4	7

Thereafter, this work was carried out following 13 experiments maintaining these parameter ranges and the resulting data are produced in Table 2. Among the three variables, the alternation of catalyst concentration was found to be the most vital, for both rapeseed oil as well as waste-corn oil. The best possible yield of biodiesel for these two substrates were obtained as 92.81% and 87.26% respectively, involving 8% w/v catalyst concentration at M:O of 13:1 and 7 h of reaction time (entry 11, Table 2).

**Table 2:** Experimental data for the optimized yield of biodiesel from rapeseed oil (a) and waste corn oil (b) in presence of SSE@GO-CuFe_2_O_4_ nanocomposite following RSM method.

Entry	A: Catalyst concentration	B: M:O	C: Time	Yield (%)^a^	
				a	b
1	5.5	13	5.5	74.22	56.27
2	5.5	10	7	79.06	73.95
3	3	13	7	73.34	59
4	8	16	5.5	80.88	58.44
5	5.5	16	4	80.37	78.33
6	8	13	4	80.06	77.78
7	3	13	4	41.91	39.83
8	5.5	10	4	73.63	59
9	5.5	16	7	74.90	72.53
10	8	10	5.5	92	85.17
11	8	13	7	92.81	87.26
12	3	16	5.5	73.29	56.97
13	3	10	5.5	56.47	56.27

^a^Reaction temperature 65 °C, Isolated yield.

The set of optimized parameters obtained by RSM analysis was further interconnected to coefficients of interactions, linear and quadratic effects by Analysis of variance (ANOVA). The probability values for correlation coefficients and variable parameters for each model has been shown in Tables 3 and 4 for rapeseed oil and waste corn oil respectively. In Table 3 the P values for the model is 0.0005. P-values less than 0.0500 indicate model terms are significant and AA, CC, AC, BC, A^2^ are significant model terms. Values greater than 0.1000 indicate the model terms are not significant. The Model F-value of 18.30 implies the model is significant. There is only a 0.05% chance that an F-value this large could occur due to noise. The Predicted R^2^ of 0.3478 is not as close to the Adjusted R^2^ of 0.9068 as one might normally expect. Adeq Precision, which measures the signal to noise ratio, is found 15.509. The ratio greater than 4 is highly desirable. This model indicates an adequate signal and can be used to navigate the design space^75^.

**Table 3:** Analysis of variance (ANOVA) for the biodiesel production from rapeseed oil over SSE@GO-CuFe_2_O_4_ nanocomposite.

Source	Sum of square	df	Mean square	F-value	P-value	Remarks
Model	2782.28	9	309.14	18.30	0.0005	Significant
A-A	1742.27	1	1742.27	103.16	< 0.0001	
B-B	68.80	1	68.80	4.07	0.0833	
C-C	389.20	1	389.20	23.04	0.0020	
A-B	4.12	1	4.12	0.2440	0.6365	
A-C	157.00	1	157.00	9.30	0.0186	
B-C	155.00	1	155.00	9.18	0.0191	
A^2^	197.71	1	197.71	11.71	0.0111	
B^2^	1.29	1	1.29	0.0761	79.06	
C^2^	56.33	1	56.33	3.34	0.1106	
Residual	118.22	7	16.89			
Lack of fit	118.22	3	39.41			
Pure error	0.0000	4	0.0000			
Core Total	2900.51	16				

R-Squared 0.96.

Adj R-Squared 0.9068.

Pred R-Squared 0.3478.

Adeq Precision 15.509.

**Table 4:** Analysis of variance (ANOVA) for the biodiesel production from waste corn oil over SSE@GO-CuFe_2_O_4_ nanocomposite.

Source	Sum of square	df	Mean square	F-value	P-value	Remarks
Model	177.80	3	59.27	7.14	0.0044	Significant
A-A	138.28	1	138.28	16.65	0.0013	
B-B	20.64	1	20.64	2.49	0.1389	
C-C	18.88	1	18.88	2.27	0.1555	
Residual	107.93	9	11.99			
Lack of fit	118.22	3	39.41			
Pure error	0.0000	4	0.0000			
Core total	285.73	16				

R-Squared 0.62.

Adj R-Squared 0.53.

Pred R-Squared 0.21.

Adeq Precision 8.25.

In Table 4, The Model F-value of 7.14 and P-values less than 0.0500 implies that the model is significant. There is only a 0.44% chance of F-value to deviate due to noise. Here AA is a significant model term. Values greater than 0.1000 indicate the model terms are not significant. Here also, the Predicted R^2^ of 0.2094 is not as close to the Adjusted R^2^ of 0.5351 and may indicate a large block effect or a possible problem with your model and/or data. But the Adeq Precision ratio is 8.247 (> 4.0) indicating an adequate signal. This model also can be used to navigate the design space.

**Figure 12:**
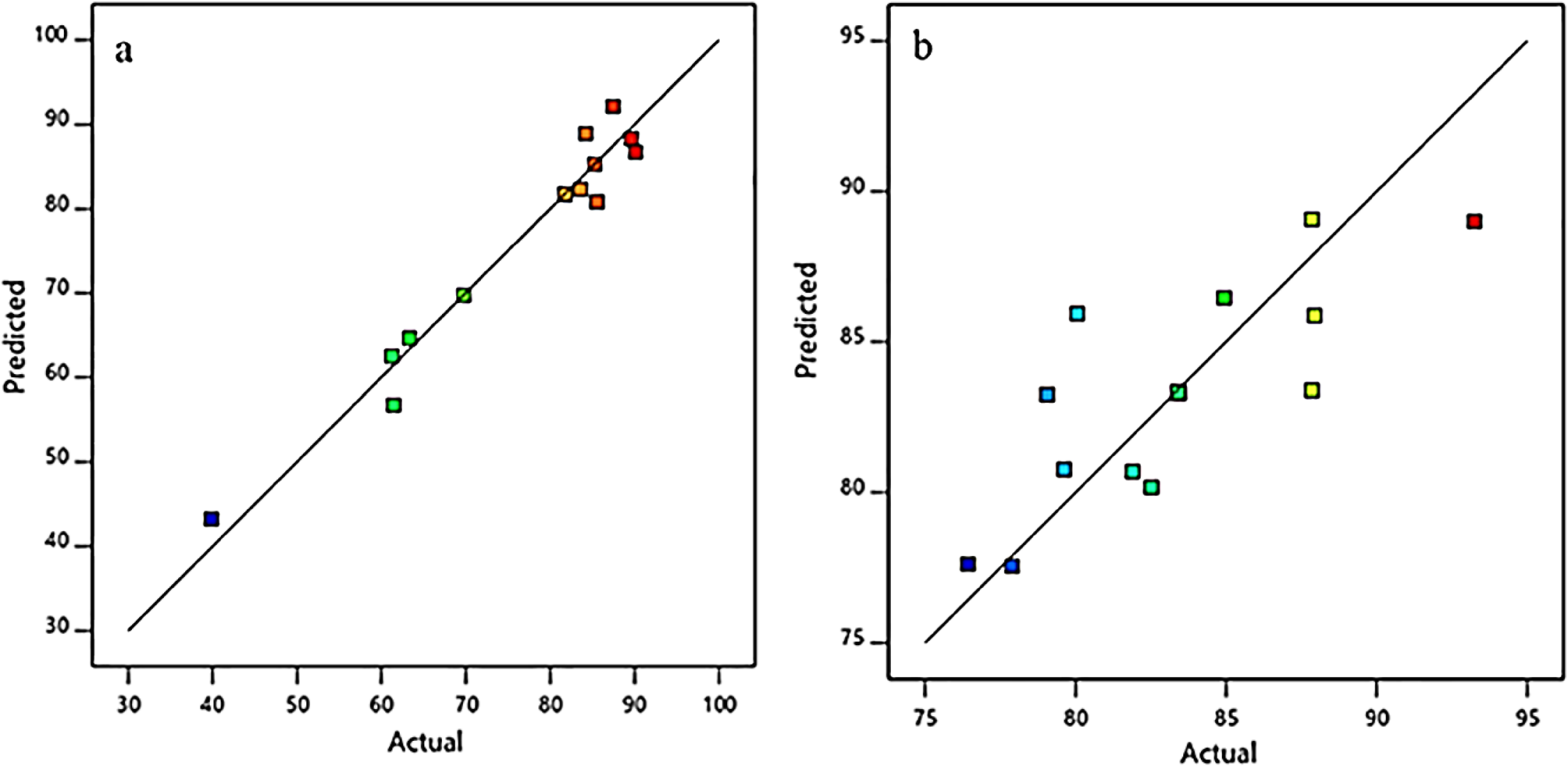
The comparison of predicted value against the actual value by the RSM in rapeseed oil (**a**) and waste corn oil (**b**).

Subsequently, the regression model developed through ANOVA was justified by plotting the experimental or actual values of the parameters against the predicted values. Evidently, the parameter values are in close agreement for both rapeseed oil and waste corn oil (Fig. 12). This signifies that the regression model can explain the connection between independent variables and the response, biodiesel yield. All the values are leaning towards a mean straight line and the chance of errors are insignificant.

**Figure 13:**
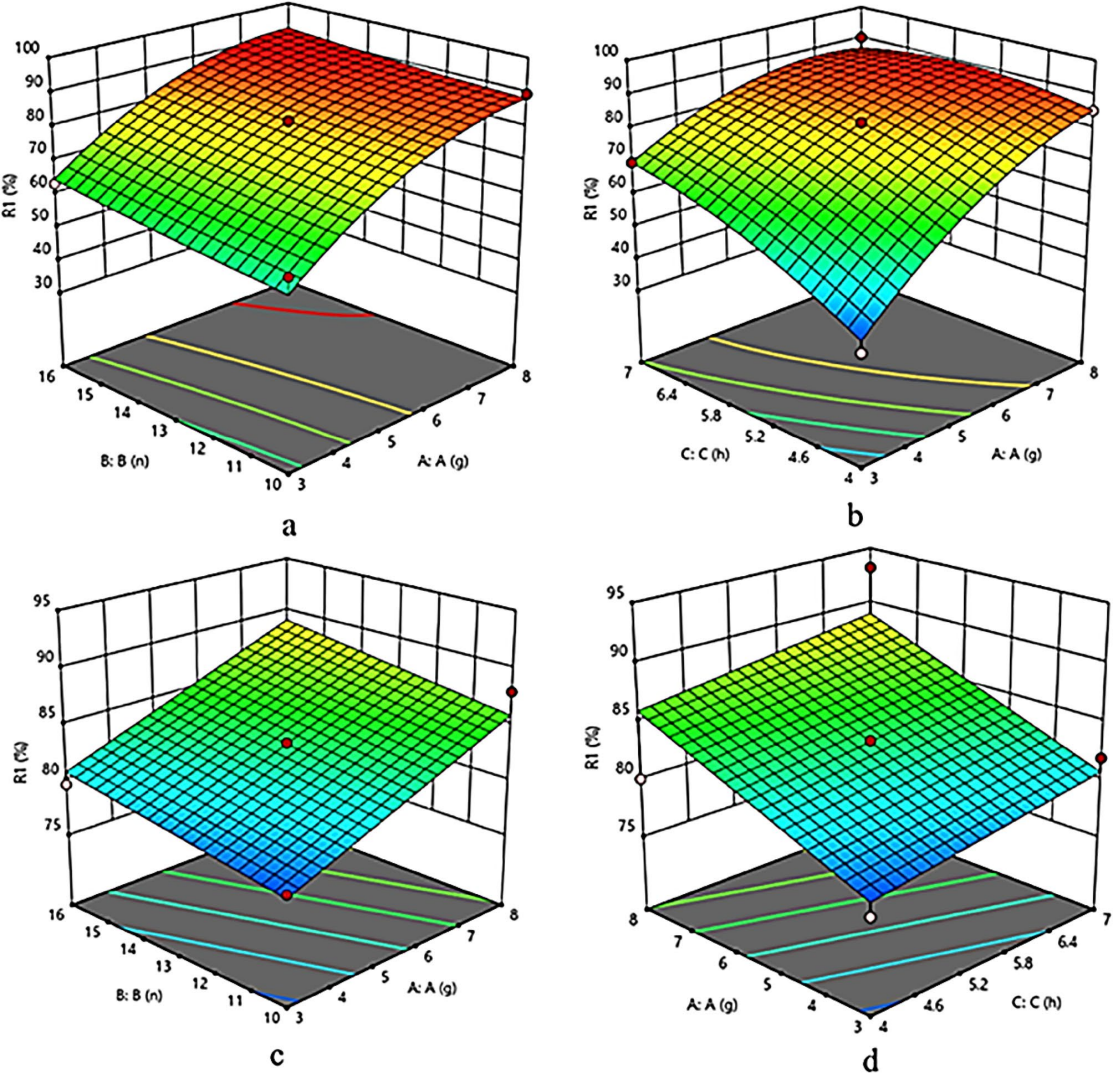
3D response surface plots of catalyst concentration and M:O for rapeseed oil (**a**); catalyst concentration and time for rapeseed oil (**b**); catalyst concentration and M:O for waste corn oil (**c**); catalyst concentration and time for waste corn oil (**d**).

In order to explain the effect of independent variables on the biodiesel yields, the 3-D response surface contour plots are drawn with the help of RSM. In the contour plot Fig. 13a,c, the optimization of biodiesel yield (%) has been displayed with variable catalyst concentration and methanol/oil ratio for rapeseed oil and waste corn oil respectively, over a constant time period of7 h. In Fig. 13a the productivity increases as a hyperbolic plane and 13c as a linear plane with increase in catalyst concentration from 3% w/v to 8%w/v and decrease in the M:O from 16:1 to 10:1. The best value was obtained at catalyst concentration of 8%w/v and the M:O of 13:1 in both the substrates. Similarly, Fig. 13b,d represents the optimization of biodiesel yield (%)with different catalyst concentration and time for rapeseed oil and waste corn oil respectively, over a constant methanol/oil ratio of 13:1. Here also in Fig. 13b,d, the % biodiesel yield increases as a hyperbolic and linear plane with increase in catalyst concentration from 3% w/v to 8%w/v and increase in time from 4 to 7 h respectively. In this two plots too yield increases with increase in catalyst concentration from 3 to 8% and increase in time from 4 to 7 h and the best result was found at 8%w/v catalyst concentration and 7 h of reaction time.

This work has been compared with the previously reported ones and been documented in Table 5. This clearly demonstrates that our work is greatly advantageous in terms of high efficiency, eco-sustainability, easy magnetic separation and also involvement of green material in the novel catalyst in contrast to previously reported works. Being a heterogeneous catalyst, reusability of the material is very important. Interestingly, the catalyst was used three times in succession without considerable loss in its reactivity. After the 4th run the productivity went down abruptly, might be due to leaching of active site from the nanocomposite surface.

**Table 5:** A comparison of catalytic performance for the transesterification reaction.

Entry	Catalyst (mol%)	Oil	Alcohol	Yield (%)	Refs
1	Na/NaOH/ɤ-Al_2_O_3_	Palmitic	Methanol	11	77
2	M. miehei (Lipozyme IM_60_)	Tallow, Soybean, Rapeseed	Methanol	19.4	78
3	ZrO_2_ supported La_2_O_3_ catalyst	Sunflower oil	Methanol	84.9	79
4	P. Juorescens	Sunflower	Methanol	3	80
5	P. Cepuciu (Lipase PS-30)	Palm kernel	Methanol	15	81
6	Unmodified catalyst	rapeseed oil	Methanol	84.2	This work
7	SSE@GO-CuFe_2_O_4_	rapeseed oil	Methanol	92.81	This work
8	SSE@GO-CuFe_2_O_4_	waste corn oil	Methanol	87.26	this work

## Conclusions

In conclusion, we have demonstrated the engineered synthesis of a novel high surface area magnetic nanocomposite material (SSE@GO-CuFe_2_O_4_) following post-synthetic modification approach. Pistachio leaves extract was used in the green synthesis of GO-CoFe_2_O_4_nanocomposite. A spice seed of *Peganum harmala*, containing numerous organo-basic compounds were immobilized over the nanocomposite in order to exploit the basicity of the compounds as well as the high surface area of GO and magnetically retrievability of CuFe_2_O_4_. The green synthesized featured material was justified and analyzed by different physicochemical techniques like FT-IR, SEM, TEM, EDX, elemental mapping, XRD and VSM. Thereafter, the material was applied in the transesterification of rapeseed oil and waste corn oil to their methylated biodiesels with high efficacy. The basic biomolecules of spice seed extract facilitated the synthesis of biodiesels. Response surface methodology (RSM) was used for optimization of different reaction parameters like methanol to oil ratio, catalyst concentration and temperature. Based on the results, the optimum yield of the corresponding biodiesel rapeseed oil and waste corn oil were obtained at M:O of ~ 13:1 and a catalyst concentration of ~ 8%w/v at 65 °C in 7 h reaction time. The results were further validated over ANOVA and 3D response surface contour plots. The outputs obtained from our devised protocol definitely would add an impact on the use of bio-functionalized nanocatalysts towards the synthesis of bio-diesels following a sustainable pathway. In addition, the RSM methodology also would be used more frequently in the optimization of different catalytic reactions in order to have more précised results.

## Data Availability

All data generated or analyzed during this study are included in this published article.

## Abbreviations

FT-IR: Fourier-transform infrared spectroscopy
SEM: Scanning electron microscopy
TEM: Transmission electron microscopy
EDX: Energy dispersive X-ray
XRD: X-ray diffraction
TGA: Thermogravimetric analysis
VSM: Vibrating sample magnetometer
RSM: Response surface methodology
ANOVA: Analysis of variance
